# Platelets in the NETworks interweaving inflammation and thrombosis

**DOI:** 10.3389/fimmu.2022.953129

**Published:** 2022-08-01

**Authors:** Ann-Katrin Wienkamp, Luise Erpenbeck, Jan Rossaint

**Affiliations:** ^1^ Department of Anesthesiology, Intensive Care and Pain Medicine, University Hospital Münster, Münster, Germany; ^2^ Department of Dermatology, University Hospital Münster, Münster, Germany

**Keywords:** platelets, neutrophils, neutrophil extracellular traps, NETs, immunothrombosis, COVID-19

## Abstract

Platelets are well characterized for their indispensable role in primary hemostasis to control hemorrhage. Research over the past years has provided a substantial body of evidence demonstrating that platelets also participate in host innate immunity. The surface expression of pattern recognition receptors, such as TLR2 and TLR4, provides platelets with the ability to sense bacterial products in their environment. Platelet α-granules contain microbicidal proteins, chemokines and growth factors, which upon release may directly engage pathogens and/or contribute to inflammatory signaling. Additionally, platelet interactions with neutrophils enhance neutrophil activation and are often crucial to induce a sufficient immune response. In particular, platelets can activate neutrophils to form neutrophil extracellular traps (NETs). This specific neutrophil effector function is characterized by neutrophils expelling chromatin fibres decorated with histones and antimicrobial proteins into the extracellular space where they serve to trap and kill pathogens. Until now, the mechanisms and signaling pathways between platelets and neutrophils inducing NET formation are still not fully characterized. NETs were also detected in thrombotic lesions in several disease backgrounds, pointing towards a role as an interface between neutrophils, platelets and thrombosis, also known as immunothrombosis. The negatively charged DNA within NETs provides a procoagulant surface, and in particular NET-derived proteins may directly activate platelets. In light of the current COVID-19 pandemic, the topic of immunothrombosis has become more relevant than ever, as a majority of COVID-19 patients display thrombi in the lung capillaries and other vascular beds. Furthermore, NETs can be found in the lung and other tissues and are associated with an increased mortality. Here, virus infiltration may lead to a cytokine storm that potently activates neutrophils and leads to massive neutrophil infiltration into the lung and NET formation. The resulting NETs presumably activate platelets and coagulation factors, further contributing to the subsequent emergence of microthrombi in pulmonary capillaries. In this review, we will discuss the interplay between platelets and NETs and the potential of this alliance to influence the course of inflammatory diseases. A better understanding of the underlying molecular mechanisms and the identification of treatment targets is of utmost importance to increase patients’ survival and improve the clinical outcome.

## Graphical Abstract

The impact of platelet-NET interplay in the background of inflammatory diseases.

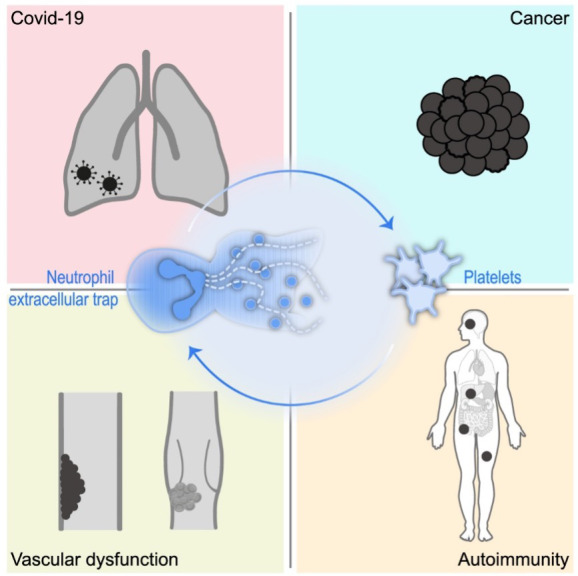

## 1 Introduction

Platelets are anucleate, small blood cells derived from megakaryocytes residing in the bone marrow and proved indispensable for ensuring vascular integrity at the site of injury. A single megakaryocyte secretes thousands of new platelets into the bloodstream through fragmentation from pseudopodal elongations named proplatelets (1). Platelets express a plethora of immune receptors on their surface, rendering them capable to probe the surrounding milieu for signs of pathogen invasion and inflammation. For instance, expression of toll-like receptors (TLRs) in platelets was confirmed for TLRs 1,2 and 6, as well as TLRs 3, 4, 5, 8 and 9 and may vary under different health and disease conditions. TLR engagement leads to platelet aggregation, increase in surface P-selectin expression, degranulation and formation of aggregates with other immune cells, such as monocytes and neutrophils (2). Activation of platelets initiates intracellular signaling cascades, resulting in the activation of surface integrins, platelet granule content release, sustenance of the coagulation cascade and the recruitment of other platelets and immune cells into a forming clot (3, 4).

Neutrophils are the most abundant innate immune cells in the circulation and are also the first to be recruited into the inflamed tissue. Packed with a diverse weaponry, their main task is to fight invading pathogens and to prevent dissemination. Neutrophil extracellular trap (NET) formation, or NETosis, which was first observed by Takei et al. and later examined in greater detail by Brinkmann and colleagues, is only one of their abilities to prevent pathogen spreading by entrapping and killing them (5, 6). Like any other of the neutrophil cell-intrinsic effector functions, NETs can be seen as a double-edged sword. NETs are mainly comprised of DNA, mixed with histones and antimicrobial proteins, expelled by the neutrophil into the extracellular space to serve host defense. However, especially histones have been demonstrated to damage endothelial and epithelial cells (7). Granule enzymes, such as myeloperoxidase (MPO), neutrophil elastase (NE) and cathepsin G, are highly bactericidal but may also damage bystander tissue (8, 9). A special role here is attributed to platelets, which were repeatedly shown to induce and promote NETosis. Additionally, NETs seem to be positioned at the interface of immunity and thrombosis, acting as a scaffold for platelet aggregation and participating in the coagulation cascade (10). From a physiological perspective, this serves as an important mechanism, where platelets and neutrophils combine forces to prevent pathogen dissemination (11), but it is also connotated with prolonged inflammation and thrombotic dysregulation, like for example in cardiovascular disease and autoimmunity.

In this review, we will take an in-depth look into how platelets activate neutrophils for NET formation, how NETs trigger platelet activation, as well as coagulation and how this intimate interplay may influence the course of disease during inflammatory events. Finally, we will discuss possible therapeutic approaches targeting platelet-NET interplay and point out future perspectives for this research field.

## 2 Platelets stimulate neutrophils for NETosis

The physical interaction between platelets and neutrophils is most importantly mediated by platelet P-selectin and neutrophil P-selectin glycoprotein ligand 1 (PSGL-1), which induces the activation of cell surface integrins on neutrophils, such as LFA-1 and Mac-1, offering the possibility to transition to tighter bond formation between the two players (12). Receptor-ligand mediated platelet-neutrophil crosstalk and subsequent activation is reviewed extensively in the literature and shall therefore not be the main focus of this review (4, 13).

### 2.1 Platelet-neutrophil interaction leading to NET formation

**Figure 1:**
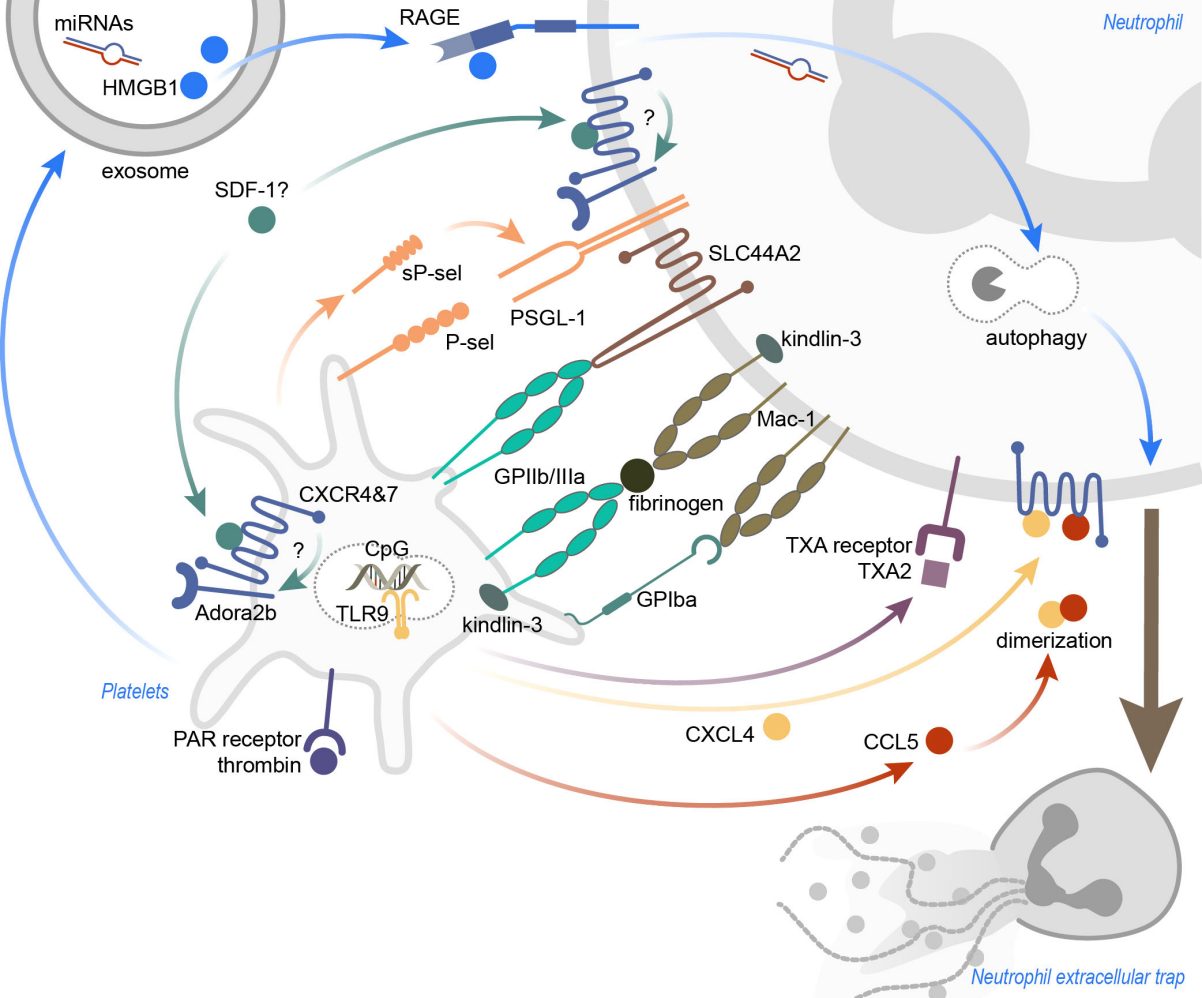
Inflammation-induced platelet-mediated NET formation. The mechanisms leading to platelet-induced NET formation are highly dependent on the surrounding inflammatory conditions. Receptor-ligand binding, as well as soluble mediators, were identified to promote platelet-induced NETosis. Ligation of (soluble) P-sel and neutrophil PSGL-1 was shown to result in NET formation, and also activation of neutrophil Mac-1. SLC44A2 was recently identified as a mechanotransducer of NETosis. CXCR4 and CXCR7, expressed on platelets and neutrophils, may be important to induce NETosis, and functional Adora2b signaling seems indispensable for this. Thrombin-activated platelets not only recruit neutrophils into the inflamed tissue, but also stimulate neutrophils to form NETs. Platelet exosomes, carrying HMGB1 and miRNAs, induce NETosis in a RAGE- and autophagy-dependent manner. TLR9 activation via CpG oligonucleotides lead to the discharge of platelet CXCL4, but CXCL4 also heterodimerizes with CCL5 to induce NETs. Lastly, blocking the neutrophil thromboxane receptor diminished NETosis. CXCR, C-X-C motif chemokine receptor; CXCL4, C-X-C motif ligand 4; CCL5, CC-chemokine ligand 5; GP, glycoprotein; HMGB1, high mobility group box 1; miRNAs, microRNAs; PAR, protease-activated; P-sel, P-selectin; RAGE, receptor for advanced glycation end products; SDF-1, stromal cell-derived factor 1; sP-sel, soluble P-selectin; TLR9, toll-like receptor 9; TXA, thromboxane A; TXA2, thromboxane A_2_.

**Figure 2:**
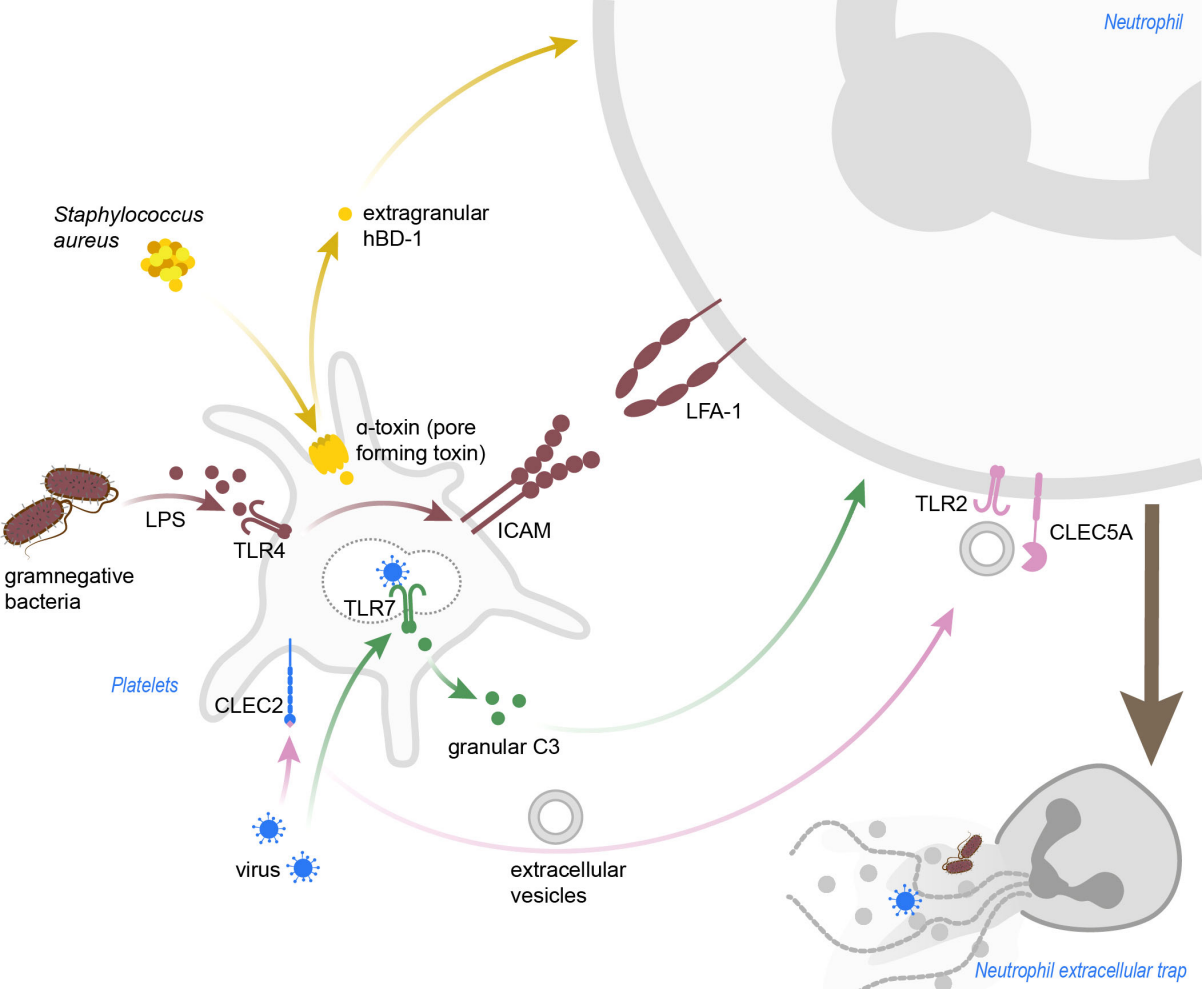
Pathogen-induced platelet-mediated NET formation. *Staphylococcus aureus* α-toxin induces pore formation in platelets, which then release hBD-1. Gramnegative bacteria and LPS activate platelets in a TLR4-dependent manner, and blocking of LFA-1 in this background abrogated NETosis in neutrophils. Dengue viruses can be sensed by platelets via CLEC2, which leads to shedding of extracellular vesicles promoting NETosis in a TLR2- and CLEC5A-dependent manner. Inlfuenza A virus are recognized via TLR7, and granular C3 then induces NETosis in neutrophils. C3, complement C3; CLEC2, C-type lectin-like receptor 2; CLEC5A, C-type lectin domain containing 5A; hBD-1, human β-defensin 1; ICAM, intercellular adhesion molecule; LFA-1, leukocyte function-associated antigen 1; LPS, lipopolysaccharide; TLR, toll-like receptor.

Over the past 20 years, the scientific community has come to a fundamental understanding of how platelets possibly stimulate neutrophils to cast NETs. There are mainly two mechanisms which are described, that can lead to platelet-induced NETosis: first, as the result of direct cell-cell contact and receptor-ligand interaction between platelets and neutrophils, respectively (14), or second, initiated through platelet-derived soluble mediators (15, 16) (**Figure 1**). However, a role for the synergistic action of both mechanisms must not be neglected (17). Additionally, some pathogens were shown to directly act on platelets, which then resulted in the downstream formation of NETs (**Figure 2**). One thing that all reports of *in vitro* experiments with platelets have in common is the necessity to activate the platelets with physiological stimuli, as non-activated platelets generally fail to induce NET formation (14, 18–20). One of the first reports by Clark et al. in 2007 not only showed that activated platelets can induce NETs, but also indicated a major role for platelet TLR4 in NET formation during sepsis (18). Platelet TLR4 engagement *via* its agonist LPS resulted in the activation of platelets, enhanced binding of platelets to neutrophils, neutrophil degranulation and ultimately NET formation. The observed NETs were formed under flow conditions only after coincubation of human neutrophils with LPS- or septic plasma-stimulated platelets. Moreover, the NET structures were able to capture and kill bacteria, demonstrating high physiological relevance for platelet-mediated NET formation in septic conditions (18). This TLR4-platelet axis dependency was also demonstrated by Liu et al., where LPS stimulation *in vitro* alone was shown to be insufficient, and only combined with platelets induced NETs (21). Albeit this remains arguable, as there are also reports where LPS induced NETosis in platelet-free conditions (22). Platelet-neutrophil interaction also leads to NET formation in a murine model of LPS-induced endotoxemia, and this was highly dependent on β_2_ integrins (14). More specifically, blocking LFA-1 in neutrophils resulted in the abolishment of NET formation *in vitro* and concomitant with this, *LFA-1* ^-/-^ mice displayed significantly reduced trapping of bacteria, in accordance with higher bacterial dissemination after peritoneal infection. Surprisingly, the formed intravascular NETs in liver sinusoids were even more important and efficient in capturing bacteria than Kupffer cells (14).

Recently, evidence for the involvement of the pro-inflammatory receptors CXCR4 and CXCR7, both expressed on platelets and neutrophils, in NET formation was presented (23–25). During the course of inflammation, the expression of these receptors is elevated in platelets and neutrophils and promotes the formation of platelet-neutrophil complexes (23). Interestingly, blocking either the CXCR4 or CXCR7 receptor significantly lowered the expression of P-selectin, PSGL-1 and L-selectin on platelet-neutrophil aggregates after initiation of sepsis (23). A functional adenosine receptor A2B signaling may be crucial here, since *Adora2b^-/-^* mice failed to show the effects observed after CXCR4 and CXCR7 inhibition. Further, the expression of NET-related genes, such as MPO and PAD4, was lowered after CXCR4 and CXCR7 blocking and here also, defective Adora2b signaling in contrast lead to elevated levels of genes involved in NETosis (MPO, PAD4 and NE) (23).

Platelet glycoproteins prove to be important in sterile platelet-mediated NET formation. In a mouse model of transfusion-related acute lung injury (TRALI), injection with tirofiban, a GPIIb/IIIa receptor antagonist, substantially reduced NET formation and protected mice from the consequences of TRALI (19). Human platelets were able to induce NETosis *in vitro* after stimulation not only with LPS, but also with the protease-activated receptor-1 (PAR-1) agonist, thrombin receptor activator peptide (TRAP), and thrombin, indicating a possibility for platelets to induce NETs in a sterile environment, irrespective of TLR engagement (19). Blocking the Raf-MEK-Erk pathway also reduced NET formation, which indicates an NADPH oxidase (NADPHox)-dependent platelet-induced NET formation in sterile inflammation (19). Similarly, it was shown that in ventilator-induced lung injury (VILI), NETosis also depends to a high extent on platelets and lung injury can be prevented by depleting platelets or preventing NET formation, respectively (17). Opposed to the findings of McDonald et al. regarding platelet-driven NETosis in sepsis, LFA-1 does not seem to play a role in sterile NETosis, but blocking Mac-1 significantly reduced NET formation. Moreover, genetic ablation of DAP12 or FcRγ, as well as inhibition of G-protein coupled receptor (GPCR) signaling (through pertussis toxin) prevented the formation of NETs, thus showing that integrin- and GPCR signaling may be required for this step (17).

The most important receptors mediating platelet-neutrophil binding are certainly P-selectin and its ligand, PSGL-1. P-selectin is stored in platelet alpha-granules and these granules fuse with the plasma membrane after stimulation, leading to increased P-selectin surface expression on platelets (26). Thus, it does not seem surprising, that the disruption of the P-selectin/PSGL-1 interaction is able to inhibit NETosis elicited by activated platelets (27, 28). More interestingly, Etulain and colleagues showed that soluble P-selectin seems to play a role in priming neutrophils for NETosis. Neutrophils from mice, overexpressing a P-selectin without the cytoplasmic domain (leading to enhanced shedding), were more eager in casting NETs in response to a co-stimulus (platelet activating factor, PMA or ionomycin) in comparison to the control (28). Furthermore, in a rat model of endocarditis, *Streptococcus mutans*-activated platelets promoted NET formation through heightened expression of P-selectin (27). Likewise, blocking the interaction of P-selectin with PSGL-1 abrogated NET formation. IgG-coated *S. mutans* activated platelet Src family kinases, Syk, PI3K and p38 MAPK signaling and this led to an increase in P-selectin surface expression. In accordance with the work from Etulain et al., soluble recombinant P-selectin in combination with *S. mutans* was sufficient to trigger NETosis, highlighting again the importance of P-selectin binding to PSGL-1 as a putative costimulatory signal to transduce NET signaling (27). Kindlin-3 is expressed in neutrophils and platelets and is known to support integrin-mediated activation of neutrophils (29). Leukocyte adhesion deficiency type III is, amongst others, associated with mutations in kindlin-3 (30). Mice lacking an intact kindlin-3 integrin-interaction domain showed disrupted NET formation in a murine sepsis model (31). Moreover, in a mouse model of deep vein thrombosis (DVT), platelets lacking kindlin-3, as well as antibody-mediated blocking of platelet GPIIb/IIIa resulted in compromised NET release, indicating the necessity of functional integrin signaling in platelets to induce NETosis. This may be due to reduced or failed platelet activation, since integrin signaling is crucial for immune cell activation. Inversely, absence of kindlin-3 in neutrophils promoted NET formation, suggesting kindlin-3 as a molecular brake in neutrophils (32). Taken together, kindlin-3 seems to play cell-type specific roles for platelet-induced NETosis, and this further supports the requirement of functional integrin-signaling in both platelets and neutrophils with respect to NET formation. A new hypothesis from Constantinescu-Bercu et al. suggests a specific mechanosensitive NETosis signaling in neutrophils, transmitted by the 10 transmembrane-spanning receptor SLC44A2. In a flow chamber model, the authors demonstrated that the von Willebrand factor (VWF)-GPIbα interaction primes platelets under flow conditions, resulting in activation of the platelet integrin GPIIb/IIIa on the cell surface and second, binding of activated platelet GPIIb/IIIa to neutrophil SLC44A2 leads to NADPHox- and Ca^2+^-dependent NET formation. The importance of the SLC44A2 receptor in hemostasis is indicated by the discovery that humans with mutations in the *SLC44A2* gene have a diminished risk to develop DVT (33). The downstream pathways of mechanosensitive SLC44A2-induced NETosis or rather how receptor-ligand interaction leads to Ca^2+^ influx and NADPHox activation remain mostly undefined until now.

### 2.2 The effect of platelet-derived soluble mediators on NET formation

The crosstalk between platelets and neutrophils not only depends on receptor-ligand binding, but is also in a large part influenced by soluble mediators. The dispensability of receptor-ligand binding between platelets and neutrophils in the context of NET formation still remains debatable to date. Using a trans-well setup, incubating platelets from ANCA-associated vasculitis (AAV) patients with neutrophils separated through a membrane, the platelets still managed to induce NETs in neutrophils (15). Interestingly, platelets from AAV patients not only display higher P-selectin surface expression, but also raised TLR9 expression, indicating a certain flexibility in TLR-homeostasis in platelets. In accordance, CpG oligodeoxynucleotides, a TLR9-agonist, induced CXCL4 release from AAV platelet granules and this ultimately resulted in NETosis (15). Platelet CXCL4 was also shown to induce NETosis in VILI, after heterodimerization with CCL5 (17). The CXCL4-CCL5 heterodimer acts pro-inflammatory by promoting neutrophil adhesion (34). Here, disrupting CCL5 and CXCL4 dimerization with a specific blocking peptide effectively prevented NET formation (17). This was also shown in a mouse model of myocardial ischemia/reperfusion injury, and blocking CCL5-CXCL4 dimerization additionally preserved the heart function by preventing NETosis (35). Blocking the thromboxane (TXA) receptor in neutrophils effectively lessened NETosis after stimulation with TRAP-activated platelets, speaking for an involvement of platelet-released TXA_2_ in NET formation after platelet activation (19). Moreover, human platelets store β-defensin 1 (hBD-1), an antimicrobial peptide, in extragranular compartments in the cytoplasm (36). *Staphylococcus aureus* α-toxin, a pore-forming toxin, was shown to induce platelet hBD-1 release. During infection with *S. aureus*, platelet-released hBD-1 effectively inhibited bacterial growth and also induced robust NET formation in human neutrophils (36).

### 2.3 Platelet-mediated NET formation in viral infections

Though it is commonly appreciated that virus infections might mostly be executed by the adaptive immunity, one cannot simply ignore the importance of innate immunity (37). For example, after myxovirus infection, circulating murine platelets adhere to neutrophils in the liver sinusoids and facilitate NET formation, which in turn protected liver cells from virus infection (37). Hantavirus and Dengue virus, both causative agents of viral hemorrhagic fever, not only elicit thrombocytopenia and activate platelets (38, 39), but also induce NET formation. Hantaviruses mediate cell entry *via* β_2_ integrins, likely inducing integrin signaling as a side effect (40). Indeed it was shown that β_2_ integrin signaling in neutrophils through hantavirus-ligation results in ROS production and NADPHox-dependent NETosis in human and murine neutrophils (40). Since the virus also activates platelets, it is tempting to speculate a role for platelets in facilitating or enhancing NET formation upon hantavirus infection, but there are currently no reports in the literature supporting this hypothesis. Dengue virus (DV)-induced platelet-mediated NET formation mainly depended on the c-type lectin receptors CLEC5A and CLEC2 (41). Whereas DV-activation of CLEC5A directly stimulated neutrophils to form NETs, the virus also activated CLEC2 on platelets, which resulted in the shedding of extracellular vesicles (EVs) promoting NET formation. NETosis was executed through EV-mediated co-activation of CLEC5A and TLR2 expressed on neutrophils. An upregulation of P-selectin on platelets was observed in DV infection *in vitro* as well as *in vivo*, yet the participation of P-selectin in triggering NETs was not examined here (41, 42). Humans infected with DV display an NADPHox-independent NETosis phenotype, as circulating NET samples in serum plasma indicated (42). Influenza A virus particles can be recognized by human platelets through TLR7 and influenza virions were demonstrated to be indeed phagocytosed by platelets (43). Virus engulfment by platelets then lead to the discharge of granular complement protein C3 and this stimulated neutrophils to release NETs (43). In a mouse model of Influenza A virus infection, platelet-recruitment into the lungs and activation clearly preceded the recruitment and activation of neutrophils (44). Targeting thrombin generation in the early stages of infection, as well as the most prominent thrombin-receptor on platelets, PAR4, significantly reduced platelet-activation, neutrophil recruitment and NET generation. This indicates a central role for thrombin not only as a pro-coagulant, but also as a pro-inflammatory signaling mediator, driving neutrophil activation and subsequent NET formation during pathogen invasion (44).

### 2.4 Platelet HMGB1 promotes NET formation

Platelet-derived exosomes carrying nucleotides, lipids and proteins might play a crucial role not only in promoting inflammation and neutrophil activation, but also during the process of NET formation (45–49). Especially high mobility group box 1 (HMGB1) protein and microRNAs (miRNAs) seem to be of importance in the context of platelet-derived exosome-induced NETosis (20, 46). HMGB1 proteins belong to the family of alarmins, which upon release may initiate inflammation in a TLR- or RAGE (receptor for advanced glycation end products)-dependent manner (50, 51). HMGB1 released from necroptotic enterocytes induced NETosis *via* the TLR4-MyD88 pathway in a mouse model of intestinal ischemia/reperfusion-induced ALI (16). Yet, HMGB1 can also be actively released from stimulated platelets and acts *via* RAGE to induce NETs in neutrophils (20). *Hmgb1^-/-^* platelets were hampered in their ability to induce NETosis and coincidingly, *Rage^-/-^* neutrophils could not be stimulated for NETosis with HMGB1. The process of NET formation after HMGB1 stimulation was also surprisingly abrogated when autophagy in neutrophils was inhibited. There are multiple publications reporting a connection between autophagy signaling pathways and NETosis, with autophagy putatively skewing neutrophils towards NET formation (20, 45, 46, 52, 53). This seems at least in parts to be dependent on the mammalian target of rapamycin (mTOR)/Akt pathway, which is known to regulate autophagy (46). Notably, two platelet-derived exosomal microRNAs, miR-15b-5p and miR-378a-3p, were shown to inhibit anti-phagocytotic mTOR/Akt signaling, which in turn enhanced NET formation (46). To date, not much is known about the impact of miRNAs on NET formation and certainly, further research is needed to examine how (exosomal) miRNAs may influence sterile and pathogen-induced NETosis.

## 3 NETs activate platelets and drive coagulation

The physiology of thrombus formation is often explained by the Virchow triad, which includes the three categories: blood flow stasis, damage of the vessel walls and hypercoagulability. Classically, occurrence of one of the three mentioned parameters may lead to clot formation. Surprisingly, NETs can function as a scaffold for thrombus formation, providing a substantial link between inflammation and thrombosis (10, 54, 55). Indeed, platelets adhere to NETs and, as a consequence, become activated. That still leaves the question, which part of the NETs actually triggers this platelet response. Heparin, a histone-removing anticoagulant, was able to dismantle NETs and also removed platelet-aggregates from the NETs as efficiently as DNase-treatment (10). This indicates that not the NETs (i.e. free chromatin), but rather incorporated proteins, such as histones, may trigger platelet aggregation and coagulation. Moreover, intact NETs often failed to induce coagulation or platelet activation *in vitro* (55, 56). The organization of DNA into chromatin, neutralizing the negative charge, completely abolished the capacity of DNA to trigger thrombin-induced coagulation, possibly explaining why intact NETs, which also consist of chromatin fibres, often fail to do so (55). On the other hand, intact NETs (with histones) were shown to trigger thrombin generation by both, platelet-independent activation of coagulation factor FXII and FXI, as well as platelet-dependent release of inorganic polyphosphate (polyP). This was enhanced after dismantling of NETs with DNase, putatively freeing NET-bound histones and other proteins (57). Platelet-derived polyP is able to activate the intrinsic pathway of coagulation in a factor FXII-dependent fashion (58). Interestingly, platelet polyP in turn also activated neutrophils for NETosis in arterial ST-segment elevation myocardial infarction (STEMI), *via* mTOR inhibition and induction of autophagy (59). The release of polyP was highly dependent on thrombin present in the vicinity of the infarct area (59), creating another road between thrombin, NETs, platelets, and polyP leading to thrombus formation. In the end, the procoagulant capacity of NETs as a whole remains a matter of debate, though it is worth mentioning that most of these studies were carried out *in vitro* and the pathophysiological conditions found in animal models may provide deviating results. Furthermore, neutrophil-derived microparticles (MPs) were shown to adhere to NETs in abdominal sepsis in mice and promote thrombin generation (60). These MPs were released after PMA-induced NETosis *in vitro* and adhered to histones on the NETs *via* phosphatidylserine. Interestingly, MP formation was not connected to PAD4-driven NET formation pathways and must therefore occur, although inducible by PMA, independently of NET formation. Moreover, these MPs had the capacity to bind Factor XII and induce thrombin generation *via* the intrinsic pathway of coagulation, possibly facilitating (a more localized) thrombus formation (60).

**Figure 3:**
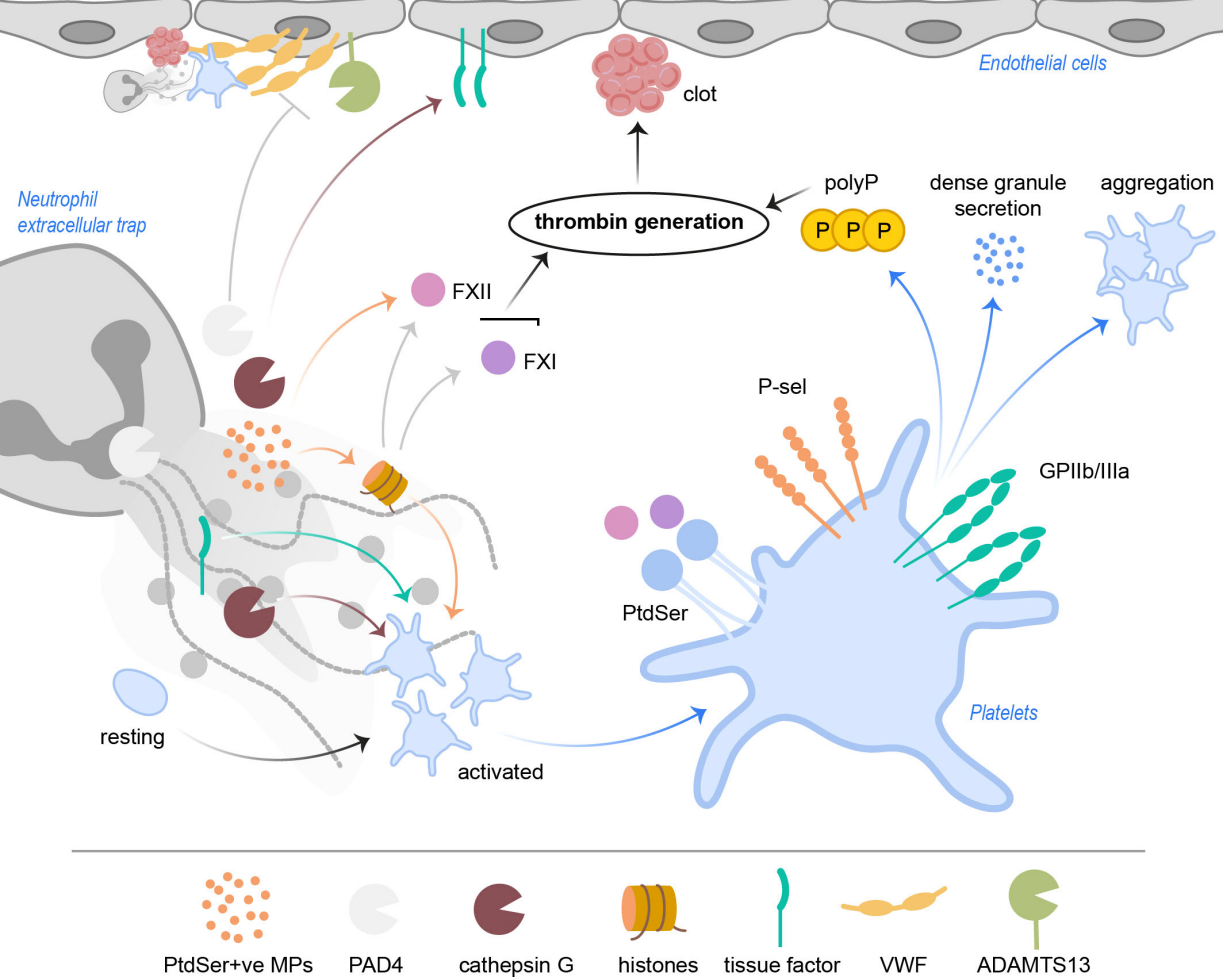
NETs activate platelets and drive coagulation. Cathepsin G and histones, especially H3 and H4, but also tissue factor directly activate platelets. PtdSer+ve microparticles in NETs provide a negatively charged surface, ideal for accumulation of coagulation factors. NETs with histones activate FXII and FXI, which results in thrombin generation. FXII and FXI also bind PtdSer exposed on platelets. Activated platelets increase P-selectin and GPIIb/IIIa activity, resulting in increased aggregation as well as signaling events leading to release of polyP and dense granule secretion. Ultimately, subsequent thrombin generation leads to clot formation in the vasculature. PAD4 was shown to inhibit ADAMTS13 activity, which hampers cleavage and removal of VWF strings. NET cathepsin G triggers tissue factor production in endothelial cells, further driving coagulation. ADAMTS13, a disintegrin and metalloproteinase with thrombospondin type-1 motif-1, member 13; FXII, factor XII; FXI, factor XI; GP, glycoprotein; MPs, microparticles; PAD4, peptidylarginine deiminase 4; PtdSer, phosphatidylserine; P-sel, P-selectin; polyP, inorganic polyphosphate; VWF, von Willebrand factor.

Triggering of platelet activation and aggregation *in vitro* was shown for NET proteins histones H3 and H4, as well as cathepsin G (**Figure 3**) (10, 54, 61). Histones H3 and H4 may thereby act *via* TLR2 and TLR4 pathways (61). Furthermore, histones (in particular H3 and H4) trigger activation of the nod-like receptor protein (NLRP)-3 inflammasome signaling and caspase-1 cleavage in platelets, which may also lead to subsequent thrombus formation (62). Stimulation of platelets with NETs and histones also leads to an increase in surface expression of P-selectin, as well as phosphatidylserine (PtdSer) exposure on the cell surface of platelets (54, 62). This is an important clue as to how NETs may trigger platelet-driven coagulation, since especially PtdSer exposure on the plasma membrane was demonstrated to facilitate the accumulation of coagulation factors on the negatively charged phospholipids (63) and thus thrombin generation. The platelet adenosine diphosphate (ADP)-receptor P2Y_12_, as well as adhesion receptor GPIIb/IIIa were shown to play a role in platelet activation after stimulation with NETs (54). Syk-mediated tyrosine-phosphorylation was increased after platelet stimulation with NETs, leading to an increase in phosphorylated downstream Akt and Erk1/2. Blocking Syk-induced phosphorylation leads to reduced platelet aggregation, dense granule secretion, P-selectin and activated GPIIb/IIIa expression (54). Additionally, NADPHox1 was demonstrated to be responsible for platelet dense granule secretion after NET stimulation (54). This demonstrates the broad diversity of signaling pathways which NETs can stimulate in platelets.

### 3.1 VWF links NET-formation and coagulation

VWF is able to bind histones *via* its A1 domain (64), pointing to another interface between NET formation and coagulation. Beyond, histamine-stimulated endothelial cell-released VWF fibres were shown to bind DNA, as well as NETs, directly *via* the aforementioned A1 domain, without the involvement of additional proteins such as histones (65). In this *in vitro* experiment, the platelets were unable to bind to VWF, because of the competition between DNA and platelet GPIbα for the VWF A1 domain (65). A disintegrin and metalloproteinase with thrombospondin type-1 motif-1, member 13 (ADAMTS13) usually cleaves formed VWF-platelet strings into small fragments, clearing them from the endothelial cell surface and thereby reducing thrombogenicity (66). Additionally, cleaving VWF by ADAMTS13 has been shown to reduce leukocyte migration to sites of injury (67). Experiments showed that occupation of VWF by DNA slightly reduced the cleavage capacity of ADAMTS13 by potentially changing the electric charge around the A2 domain (cleavage site of ADAMTS13) *in vitro*, however VWF removal did not seem much affected by this (65). It is worth mentioning though, that the authors used protein- and RNA-free DNA for their experiments, thus using intact NETs may provide different results regarding VWF-removal. Peptidylarginine deiminase 4 (PAD4), an enzyme involved in histone citrullination and chromatin decondensation, important steps during NETosis, promotes DVT in mice (68). It is now known that PAD4 also negatively affects ADAMTS13 activity (69). Injecting recombinant human PAD4 into mesenteric venules in wildtype mice lead to a significant increase in VWF-platelet strings and also accelerated stable thrombus formation when mice venules were simultaneously treated with ferric chloride. Apparently, PAD4 citrullinates and inactivates ADAMTS13, which renders the enzyme unable to cleave VWF-platelet strings, possibly being the underlying cause of the observed pro-thrombotic effect of PAD4 (68, 69).

### 3.2 NETs induce tissue factor production and cleave Thrombospondin-1

Tissue factor (TF) is an important initiator of coagulation (70). NETs were shown to induce TF production in endothelial cells *via* cathepsin G and IL-1α, in line with increased clotting (71). Moreover, bioactive TF is localized in NETs from sepsis patients’ neutrophils (53). Neutrophils from healthy donors incorporated intracellular TF into the NETs after simultaneous incubation with pro-inflammatory TNF-α, IL-1β and G-CSF, and this was greatly increased after incubation of neutrophils with the cytokine mix in the presence of *Escherichia coli.* Moreover, only TF-bearing NETs were able to increase thrombin generation and PAR-1 signaling in platelets, connecting inflammation and thrombosis in septic conditions. Interestingly, autophagy seems to present an alternative pathway, through which proteins can be incorporated into NETs, as was indicated here for TF and HMGB1 (53). This at the same time argues for a heterogeneity in NETs themselves, which might differ in their composition following divergent NET triggers.

Thrombospondin-1 (Tsp-1), which is mainly produced by endothelial cells and platelets, mediates platelet aggregation by bridging fibrinogen and platelet GPIIb/IIIa (72) and at the same time, exerts protective effects against ADAMTS13-mediated degradation of VWF (73). NET formation not only resulted in the controlled proteolytic degradation of the 185 kDa protein to a 160 kDa version, but also protected the smaller protein from further degradation. Notably, the 160 kDa Tsp-1 exhibits increased capability of promoting platelet adhesion and string formation under flow conditions, putatively enhancing thrombus formation (73).

## 4 The pathophysiological influence of platelet-NET interplay in inflammation

Since the beginning of investigations of putative pathophysiological influences that NETs may have on the surrounding tissue, it is clear that they exhibit a high damage potential. Human umbilical vein endothelial cells (HUVECs) and liver sinusoids suffered significant harm after platelet-mediated NET formation (14, 18), and NETs were shown to increase the permeability of primed endothelial cells (19). Moreover, many proteins woven into NETs remain active and may not only kill off pathogens, but also harm bystander cells, even after dismantling the NETs. For instance, histone H4 is toxic towards endothelial cells and smooth muscle cells, partly through the activation of TLR receptors and pore formation into the cell membrane (74, 75). Additionally, not only the direct effects that NETs DNA and proteins inflict on the environment, but also NET-induced coagulation comprises a significant danger towards the host. The ability of the immune system to trigger coagulation is manifested in the literature as immunothrombosis, and is a physiological form of thrombosis to prevent pathogen dissemination (11). Importantly, platelets and NETs have been shown to take reasonable part in the development of immunothrombosis, for instance during sepsis to prevent bacterial spreading (76). However, the misdirected platelet-NET interplay is also associated with a variety of diseases, including cardiovascular dysfunction (62, 77, 78), autoimmune diseases (79–81), cancer (52, 82, 83) and infectious diseases (44, 76), which was recently demonstrated for COVID-19. In the following section, we will elaborate the consequences of this interplay in a bit more detail.

### 4.1 Platelet-NET interplay in venous and arterial thrombosis

Platelets and neutrophils as inflammatory cells are each on their own well-defined contributors to the pathogenesis of cardiovascular diseases. However, their synergistic force regarding NET formation and thrombosis is of special scientific interest since NETs have been determined a common component of venous and arterial thrombi.

#### 4.1.1 Venous thrombosis

A strong connection between NET formation and DVT has been shown in the important and elaborate work of the Wagner group (10, 84). Murine deep vein thrombi from a venous stenosis model contain NETs in the red blood cell rich part of the thrombus, colocalizing with VWF, but not in the white, platelet-rich part. The distribution of platelets, neutrophils and NETs within the thrombus here suggested platelet-mediated recruitment and activation of neutrophils followed by NETosis and recruitment of red blood cells, tangled up in the NETs (84). Indeed, platelets proved indispensable for further recruitment of leukocytes (77). Moreover, neutrophils in the thrombus bound FXII and platelet-induced/supported NET formation led to the activation of FXII, propagating coagulation (77). Thus, NET formation in DVT may not be the initial trigger of the thrombus formation, but definitely promotes thrombosis, and platelets recruit and activate neutrophils for NETosis. A study of human venous thrombi from DVT and pulmonary embolism showed an abundance of NETs in the organizing stage of thrombus formation, which is characterized by infiltration of immune cells into the forming clot. NETs colocalized with VWF and were also located next to platelets, indicating a role for NETs in further platelet recruitment *via* NET-bound VWF into the organizing thrombus (85). Moreover, platelet HMGB1 was demonstrated to promote venous thrombosis in mice (78, 86). Interestingly, the redox form of HMGB1 seems to have an influence on its ability to induce thrombosis. Specifically, disulfide HMGB1 facilitates NETosis by binding to the neutrophil RAGE receptor. Besides, the existence of a positive feedback loop between platelets and platelet-derived HMGB1, released in an autocrine fashion, may result in additional platelet recruitment to the vessel walls (86). IL-17A contributes to DVT by activating platelets and stimulating NET formation (87). GPIIb/IIIa-kindlin-3-mediated integrin signaling has cell-type specific roles in DVT formation in mice, and kindlin-3 absence in platelets generally impaired platelet-neutrophil crosstalk resulting in abrogated NET formation, though this seems to be important only in the beginning of thrombus formation and dispensable in later stages (32). SLC44A2 as a mechanosensitive transducer of NET signaling proved important for platelet-mediated NET formation in DVT (33). In addition, another group demonstrated that murine platelets lacking Slc44a2 display impaired activation due to a failure in mitochondrial choline transport. This was responsible for the diminished thrombus formation times (88), but may also contribute to impaired stimulation of neutrophils for NET formation. Since Slc44a2 proved to be important for mitochondrial reactive oxygen species (ROS) and adenosine triphosphate (ATP) production in platelets (88), it seems likely that Slc44a2 fulfils the same function in other cells of the myeloid lineage, such as neutrophils, which may also in part explain the findings from Constantinescu-Bercu and colleagues. More work will be needed to define the role of this interesting receptor in hemostasis. Hypoxia-induced venous thrombosis relies on the proinflammatory HIF1α-NLRP-3 inflammasome-caspase-1-IL-1β axis (89). Studies show elevated serum levels of IL-1β in patients with DVT. IL-1β cleavage is mediated by caspase-1, which is activated through the NLRP-3 inflammasome. Hypoxic conditions and activation of HIF-1α may lead to the activation of the NLRP-3 inflammasome and caspase-1 cleavage. NETs and NET-derived histone proteins trigger platelet activation and promote coagulation, and this is in part dependent on the activation of the NLRP-3 inflammasome and caspase-1 by NET-bound histone H3 and H4 (62). Moreover, caspase-1 inhibition was able to reduce thrombus formation in mice, though this might have also been partly affected by the inhibition of NET-bound caspase-1 (62). Recently, a study in patients with Crohn’s disease demonstrated a link between the activation of the platelet NLRP-3 inflammasome and the observed platelet hyperactivity and increased risk of thrombosis (90), further encouraging that NLRP-3 inflammasome activation in platelets promotes coagulation.

#### 4.1.2 Arterial thrombosis

Arterial thrombosis is often initiated by the formation and/or rupture of an atherosclerotic plaque. Atherosclerosis is an inflammatory process involving endothelial cells, monocytes, and even neutrophils (91). Atherosclerotic plaque formation, if left untreated, can lead to vessel occlusion and arterial thromboembolism. The results of plaque or arterial thromboembolic vessel occlusion are manifested in peripheral artery disease (PAD), coronary artery disease (CAD) leading to myocardial infarction and intracranial atherosclerotic disease with subsequent cerebral ischemic stroke, respectively. The importance of neutrophils and NET formation in the context of atherosclerosis becomes more evident, since various studies revealed an abundance of NETs in human thrombi from the arterial vasculature (92, 93).

In patients with ST-segment elevation myocardial infarction (STEMI), NETs in thrombi retrieved from the culprit coronary artery are decorated with procoagulant TF (94). Platelet-neutrophil interaction leads to NETosis in the vicinity of plaque ruptures and neutrophils drawn from the culprit artery form NETs more frequently after stimulation. Platelets in turn, activated by the TF, are capable to stimulate neutrophils to form NETs (94). The presence of proinflammatory IL-17A in neutrophils and NETs of cardiac thrombi can promote platelet aggregation (95). Interestingly, an increase of complement C5a in the plasma of STEMI patients was recently connected to thrombus formation. C5a promoted NETosis in a STAT3- and mitochondrial ROS-dependent manner, and inhibition of C5a led to reduced thrombus formation in a murine model of ferric chloride induced left common carotid artery thrombosis (96). It remains unclear though, what initially triggers the increase in plasma C5a. Despite not being classically linked to NET formation, evidence was provided, that erythrocytes (and their fragments) in thrombi may also activate platelets and induce NET formation (97). Such interplay possibly ends in a self-sustainable positive feedback loop and aggravates thrombus formation and infarction activity. Indeed, studies show that NET formation is positively correlated with infarct size and negatively with ST-segment resolution, indicating that the presence of NET markers may predict a worse outcome in patients with myocardial infarction (98). To this end, composite biomarkers (platelet count, soluble P-selectin and NET markers) were suggested to improve prediction of the outcome after myocardial infarction (99). Acute ischemic stroke (AIS) can either be caused by thromboembolism initiated downstream of the brain vasculature, for instance atherosclerosis of the internal carotid arteries, or by cerebral vessel occlusion from intracerebral atherosclerotic plaques. Moreover, AIS patients also have an increased risk for further atherothrombotic complications and stroke recurrence (100). NETs can be found throughout the brain tissue of stroke patients and NET markers correlate with worse disease outcome (101). Furthermore, NETs were demonstrated to be a causative agent of hypercoagulability in stroke patients with internal carotid artery occlusion, indicating a contribution to the aforementioned complications and stroke recurrence (102, 103). Increased thrombin in carotid lesion site (CLS) plasma, PtdSer expression on platelets and on platelet-derived microparticles (PMPs), but also subsequent accumulation of PtdSer+ PMPs in platelet-induced NETs may be responsible for this. A role for NET-inflicted endothelial cell damage and conversion of endothelial cells into a procoagulant phenotype was proposed as well (102). Interestingly, thrombi retrieved from patients with AIS contain more platelets than those from CAD or PAD, and this is independent of the quantity of NET components in the thrombi (93), though the reason for this is currently unknown. Platelets were identified as the main source of NET-inducing HMGB1 in stroke patients (101), and another group demonstrated, that murine platelets promote arterial thrombosis *via* TLR4-dependent NETosis induction (104, 105). Hence, platelet-mediated NET formation may play a huge role in the pathophysiology of AIS.

Moreover, NETs *in vivo* may gravely enhance the thrombus stability by influencing the degradability of other components, such as VWF and fibrin (69, 106). This seems age-dependent for CAD and PAD with higher fibrin-to-histone ratios in elderly patients and may influence the decision-making regarding therapeutic approaches (93). Concomitant with this, circulating and thrombi-NETs in AIS, mostly due to activation of plasminogen activator inhibitor-1 (PAI-1) expression, display a certain therapy-resistance against treatment with tissue-type plasminogen activator (t-PA), a thrombolytic agent targeting fibrin and used in the treatment regimen of ischemic stroke (107).

### 4.2 Platelet-NET interplay in autoimmune diseases

#### 4.2.1 Systemic lupus erythematosus 

Surprisingly and opposite to the influence of NETs themselves, not quite so much is known about the role and consequences of platelet-NET interplay in autoimmune diseases. One of the best-examined examples of a pathophysiological influence of platelet-NET interplay in autoimmune diseases is sytemic lupus erythematosus (SLE). SLE is characterized through the overproduction of self-recognizing antibodies, for instance anti-nucleus antibodies (ANA) and anti-double stranded DNA (dsDNA) antibodies, which during the progression of the disease cause inflammation, vasculopathy or vasculitis (108). SLE patients have an increased risk for venous, as well as arterial thrombosis (109). Though thrombosis in SLE is described as of multifactorial origin, there is evidence of a pathogenic influence of platelets-NET interaction on coagulation. Specifically, the occurrence of antiphospholipid antibodies (aPL) and aPL syndrome poses a risk factor in the development of thrombotic complications, even during primary aPL syndrome (no simultaneous SLE diagnosis) (79). In primary aPL syndrome, neutrophils are prone to spontaneous NET formation. Anti-β_2_-glycoprotein I (anti-β_2_-GPI) managed to directly trigger NETosis *via* the TLR4/MyD88/MAPK-axis in neutrophils and these NETs expressed TF and promoted thrombin generation, possibly stimulating platelets as well (79, 110). Moreover, some aPLs, for instance anti-β_2_-GPI, were shown to induce RAGE activation and HMGB1 relocation in platelets (111). Both molecules are associated with platelet-induced autophagy-mediated NET formation (20, 46). Circulating IgG complexes may also bind to platelets *via* FcγRIIa ligation and cause subsequent thrombin hypersensitivity in SLE patients (112). Thrombin-stimulated platelets could then induce NETosis upon aggregation with neutrophils. Serum factors from Lupus patients induces Regulated in development and DNA damage responses 1 (REDD1)-directed autophagy and NETosis in neutrophils, and the resulting NETs are decorated with TF and IL17A, putatively leading to coagulation and fibrosis (80). Another risk factor for thrombosis in SLE may be the existence of so called “low density granulocytes” (LDGs). The occurrence of LDGs is elevated in SLE, as well as other autoimmune diseases (113). These neutrophils are associated with the peripheral blood mononuclear cell (PBMC) fraction after density gradient centrifugation (thus “low density”) and show an elevated capacity to form NETs (113, 114). Increased NET formation in the blood circulation of SLE patients may heighten the risk of endothelial cell damage and platelet activation, which may contribute to the elevated manifestation of venous and arterial thrombosis. SLE patients also display reduced ADAMTS13 activity, and this may also be involved in thrombotic events (115). Curiously, NETs were demonstrated to negatively influence the activity of ADAMTS13 (69), but in how far NETs and/or NET proteins mediated the reduction in ADAMTS13 activity in SLE patients has not yet been elucidated. To make matters worse, even SLE patients without development of aPL syndrome exhibit a prothrombotic state, which is reflected in activated platelets and increased platelet-leukocyte aggregates at baseline, as well as increased thrombin generation through TF in microparticles and NETs (116). Increased PtdSer exposure on platelet plasma membranes may be another reason for the observed hypercoagulability (116). NETs may also be recognized by the adaptive immunity as a self antigen and lead to the production of anti-dsDNA antibodies, which in turn activate platelets and promote thrombosis (117).

#### 4.2.2 ANCA associated vasculitis

ANCA associated vasculitis (AAV) is a systemic autoimmune disease that specifically affects the small blood vessels, thus also described as “small-vessel vasculitis”. Autoantibodies against neutrophil proteins, such as MPO and/or proteinase 3 (PR3), are a characteristic of this disease. Inflammation of the small blood vessels can affect any organ in the body and lead to significant end-organ damage. Three subtypes of ANCA vasculitis have been defined: granulomatosis with polyangiitis (GPA), microscopic polyangiitis and eosinophilic granulomatosis with polyangiitis (EGPA) (118). Neutrophils from AAV patients not only display a higher baseline NET formation, but platelets from AAV patients stimulate neutrophils for NETosis through TLR9-dependent CXCL4 release (15). Moreover, NET formation may then lead to thrombus formation (81) and general disease progression by triggering new vasculitides, since autoantigens may be abundantly present in NETs (119). Not neutrophil, but eosinophil extracellular traps (EETs) may play a larger role in EGPA than anticipated. Eosinophils, classically connotated with helminth infections and the pathology of allergies, were shown to release extracellular traps upon a variety of stimuli *in vitro*, for instance with the calcium ionophore A23187 (120). Eosinophils seem to react with excessive EET formation to sputum immunoglobulins and were also found in the sputum plugs of COPD patients (120), maybe indicating a preserved function of extracellular traps in mucosal immunity and/or parasite infections. In EGPA, EETs were detected in small-vessel thrombi, correlated with D-dimer levels and induced platelet adhesion, identifying EETs as the main miscreant in immunothrombosis in EGPA (121).

#### 4.2.3 Type 1 diabetes

An association between neutrophil infiltration, NET formation and the development of type 1 diabetes has been confirmed prior in non-obese diabetes (NOD) mouse models (122). Now, a connection between platelet-neutrophil interaction and type 1 diabetes has been presented (123). An increase of platelet-neutrophil aggregates in the circulation correlated with NET markers in NOD mice pancreas, suggesting that platelets may activate neutrophils for transmigration into islets followed by NETosis and islet destruction. Though NET formation was not as readily detected as signs of activated neutrophils (MPO+ and H3cit+) in murine NOD islets, histones and NETs were able to damage murine and human β cells and pancreatic islets *in vitro*, respectively (123). This suggests a connection between the observed progressing islet destruction *in vivo* and platelet-activated neutrophils, though the relevance of NET-induced pancreatic islet damage *in vivo* certainly needs further investigation.

### 4.3 Cancer-associated immunothrombosis

It has been appreciated for a long time that the development of cancer goes along with an increased risk of thrombosis, and this is recognized as Trousseau’s syndrome (124). A hypercoagulable state was observed in many patients with cancer diagnosis. The reasons for this are not yet fully understood and may be divided into factors that are patient-related, treatment-related and cancer-related (124). For instance, tumor-mediated secretion of procoagulant factors, such as TF (125), were shown to be responsible for cancer-induced thrombosis. Yet, malignant tumours might also induce immunothrombosis by activating platelets and/or neutrophils followed by NET formation (83).

#### 4.3.1 Tumor cells activate platelets

Tumour cells were demonstrated to activate platelets, and the underlying processes vary between different types of cancer (126). Many tumors, for instance pancreatic cancers, were shown to secrete thrombin (127), a potent activator for platelets. Moreover, COX-2 expression in cancers may lead to the downstream synthesis of TXA_2_, which not only results in tumor progression, but also platelet activation and aggregation (128). Ovarian cancer cells secrete ADP (129), which can bind to P2Y_1_ and P2Y_12_ on platelets, and this results in a positive feedback loop, with platelets releasing more ADP from dense granules. The secretion of procoagulant TF was shown for colorectal cancer cells and as part of microparticles (MPs) in circulating tumor cells (125, 130). TF initiates intravascular coagulation by acting on the extrinsic coagulation cascade, in specific on factor FVIIa, and also indirectly *via* thrombin generation and fibrin deposition on the activation of platelets (70). The tumor MPs provide a procoagulant surface due to presentation of PtdSer on the outer leaflet of the membrane (131). Podoplanin-expressing cancer cells can interact with platelets *via* CLEC-2, and this leads to platelet activation (132). Thus, the platelet-axis might be a direct way for tumours to promote thrombosis, or the tumour may act indirectly *via* platelet-mediated NET formation. There is evidence in the literature that platelets and NETs are closely connected regarding immunothrombosis in cancer. Pancreatic cancer cells, for instance, can induce NETosis and this was further increased after platelets were primed with pancreatic cancer cells (133), suggesting that activated platelets, after contact with tumor cells, may propagate NET formation. Platelet-mediated NET formation is linked to the development of VTE in high-grade glioma patients, and this is at least in part dependent on P-selectin and PSGL-1 interaction (82). NET-mediated PtdSer expression on platelets and endothelial cells, as well as increased TF expression, TAT complex and fibrin formation may be the cause for the elevated risk of VTE development here (82). Similar results were shown prior for patients suffering from colorectal cancer, and here platelet HMGB1 was a major contributor to NET formation (134). The increased risk for VTE development after glioma neurosurgery (82, 135) may be caused by the release of tumor cells into the blood circulation, which in turn may activate platelets and neutrophils and lead to the observed procoagulant state. Coincidingly, Ren et al. showed that NETs may capture circulating tumor cells (CTCs) after surgery in a platelet-dependent manner (136). Platelets, activated through the surgical stress and local inflammation, formed aggregates with the CTCs in a GPIIb/IIIa-dependent manner in the bloodstream. Here, platelet-independently formed NETs in the lungs captured the platelet-CTC aggregates and further promoted metastasis. The activation of the platelets was initiated by TLR4 and further downstream depended on the activation of ERK5, which resulted in an increased GPIIb/IIIa activation (136).

#### 4.3.2 Tumor cells induce NETs

Another way for malignant tumors to promote immunothrombosis is to induce NETs, which then in turn may activate platelets. The presence of NETs and/or NET formation was demonstrated in the background of a variety of cancers. But how exactly is cancer able to induce NETosis? The putative answer can be found mostly by taking a closer look at tumor-derived secreted vesicles and chemokines. Cancer-cell derived exosomes from female mice injected with 4T1 breast cancer cells were shown to induce NETs in neutrophils following G-CSF pre-stimulation (137). Moreover, these animals had a higher number of circulating neutrophils, going along with an increased baseline plasma DNA and MPO, both associated with NETosis (137). Tumor-derived G-CSF and IL-8 may also take part in activating neutrophils to undergo NETosis in cancer (138–140). As a matter of fact, blocking CXCR1 or CXCR2 in neutrophils upon coincubation with supernatant from various chemokine-producing carcinoma cell lines abrogated NET formation (141), which argues for the involvement of CXCR1 and CXCR2 ligands in tumor-mediated NET formation. Other tumor-secreted pro-inflammatory chemokines and cytokines may promote NET formation, yet this remains to be elucidated. NETs in turn induce a hypercoagulable state in cancer patients, as demonstrated for patients suffering from glioma, which may result in thrombotic events such as VTE or arterial microthrombosis (82, 133, 142). Furthermore, Guglietta et al. provided a link between NETs and platelets in an animal model of small intestinal tumors. Here, the mice suffer from chronic hyperactivation of coagulation and increased platelet consumption, most likely the result of complement factor C3a-activated neutrophils producing NETs (143). In accordance with the animal model, humans with small intestinal cancer often suffer from hypercoagulability and neutrophilia (143). Indeed, Armande Trousseau, the discoverer of the hypercoagulable state in cancer patients, diagnosed himself with gastric cancer after experiencing venous thrombosis along with other symptoms of the disease (144).

But what is the (patho-)physiological consequence of tumor-induced NET formation and putative subsequent thrombosis? Often, it seems that the tumor itself benefits from activating platelets or neutrophils to induce thrombosis or NETs, respectively (126). The formation of NETs is associated with disease progression in gastric cancer patients (145). NETs promoted the migration of gastric cancer cells and induced epithelial-to-mesenchymal transition by downregulation of E-cadherin and enhanced expression of vimentin in a human gastric cancer cell line, and this was abrogated after addition of DNase-1 or PAD4-inhibitor (145). Furthermore, dormant cancer cells from a primary tumor can awaken years after dissemination upon inflammatory events, and NETs were shown to take significantly part in this waking-up process (146). Neutrophil NE and matrix metalloproteinase 9 (MMP9), through proteolytic cleavage and remodeling of laminin, induced cell cycle progression in previously dormant cancer cells (146). Thus, the appearance of not only neutrophils, but especially NETs in the tumor environment seems to correlate with disease progression and worse clinical outcome. Moreover, CTCs make use of this mechanism to protect and shield themselves from the immune system, or simply as a landing hub for metastasis formation. Cools-Lartigue and colleagues demonstrated a heightened chance for circulating lung carcinoma cells to form hepatic metastases following CLP-induced microvascular NET formation, and this was abrogated when NET formation was inhibited with DNase or neutrophil elastase inhibitor (147). Interestingly, the interplay between NETs and platelets in murine small intestinal cancer directly affected tumor development, since blockade of either coagulation with low molecular weight heparin (LMWH) or disruption of C3a-R signaling reduced tumor formation (143).

Thus, understanding the interplay between platelets and neutrophils/NETs in cancer appears to be crucial to find the appropriate treatment. Taken together, the evidence provided gives the impression, that targeting platelets or NET formation in cancer in addition to cancer treatment may exert an overall beneficial effect regarding disease progression and metastasis formation.

### 4.4 The putative role of NETs and platelets in immunothrombosis of COVID-19 patients

The severe acute respiratory syndrome coronavirus-2 (SARS-CoV-2) is the causative agent of the corona virus disease 2019 (COVID-19) pandemic. The virus is transmitted *via* aerosols and infects its host through the airway epithelium. The SARS-CoV-2 spike protein binds to the angiotensin converting enzyme 2 receptor, expressed on the apical side of the lung epithelial cell membrane, and thus enters epithelial cells (148). Infection with the SARS-CoV-2 virus leads to a strong immune response, often with severe consequences for the host, such as the development of pneumonia. In more severe cases, the infection may result in acute respiratory distress syndrome (ARDS) and multiorgan failure with urgent need to be admitted to and treated in the intensive care unit (ICU).

There is a substantial body of evidence that pinpoints neutrophils as a source of disease severity, disease progression and lung injury in COVID-19 (also reviewed in (149)). Patients with severe COVID-19 have a higher neutrophil-to-lymphocyte ratio and suffer from hypercoagulability (150, 151). A higher neutrophil-to-lymphocyte ratio is often predictive of a poor outcome for the patient in several disease backgrounds, including COVID-19 (152). Neutrophils are recruited to the lungs after SARS-CoV-2 virus entry and infection (153, 154), and neutrophilia is a common symptom of COVID-19 patients (155, 156). The subsequent development of pneumonia, ARDS and multiorgan failure may be a direct outcome of several neutrophil effector functions in severely affected patients, especially NET formation. Moreover, abnormally elevated cytokine levels in infected ICU and even non-ICU patients were observed (155). Patients may present with higher plasma levels of IL-1β, IL-6, IL-7, IL-8, G-CSF, GM-CSF, IFNγ, PDGF and TNFα in comparison to healthy controls (155, 157). Specifically, IL-1β, IL-8, TNFα and G-CSF are defined NET inducers in the literature (53, 75, 158, 159), and higher levels of these cytokines, particularly in combination, may stimulate neutrophils to form NETs more readily. In fact, patients with COVID-19 show increased plasma levels of MPO-DNA complexes, cell free DNA and H3cit, all of which are indicative of NET formation (156, 160). Serum from COVID-19 patients induced NET formation in neutrophils from healthy individuals, further proving that chemokines and cytokines may be involved in NET formation (160). Additionally, blood neutrophils drawn from COVID-19 patients are often found to have internalized SARS-CoV-2 and have an increased capacity to form NETs (156). However, it is daring to state that the virus infected the neutrophils, since neutrophils do not express relevant amounts of ACE2, nor transmembrane protease serine subtype 2 (TMPRSS2), the classical SARS-CoV-2 ligands. It could be, that SARS-CoV-2 infects neutrophils by yet unidentified mechanisms, or, more likely, neutrophils act as phagocytes to clear the virus and apoptotic bodies, thereby passively taking up the viral particles. How exactly this may lead to NET formation in neutrophils remains to be elucidated. It was, for instance, demonstrated for other viruses, that neutrophil pattern recognition receptors are essential in sensing viral components and might at the same time be transducers of NETosis pathways (161), which may as well hold true in case of SARS-CoV-2. NETs were also recovered from tracheal aspirates of COVID-19 patients supported with mechanical ventilation, and from lung sections of individuals post-mortem (156). Of note, the mechanical ventilation might have contributed to NET formation here, thus ventilated non-infected patients should be used as a control instead of healthy patients.

Lung autopsies of COVID-19 patients demonstrate colocalization of NET-ting neutrophils with platelets in pulmonary blood vessels, and patients have elevated levels of platelet-neutrophil complexes (162). But not only NETs can be found in the tissue of severely ill and deceased COVID-19 patients. Microthrombi, rich in platelets, fibrin and granulocytes, were found in the vessels of lung, the kidneys, and the heart (163). Cerebral thrombi from COVID-19 patients with large vessel occlusion acute ischemic stroke show increased neutrophil infiltration (164). Curiously, despite an increase in neutrophils in the thrombi, no differences in the composition of thrombi, especially regarding NETs, between COVID-19 and control stroke patients were found (164), suggesting that neutrophils may induce immunothrombosis not exclusively *via* NET formation, or that other yet unidentified factors contribute to the thrombotic dysregulation. Interestingly, LDGs, which are associated with autoimmune diseases and shown to cast NETs more frequently than “normal density” granulocytes, were found in COVID-19 patients (165). These neutrophils were grouped into four subclusters and one of the subclusters correlated positively with increased NET markers (circulating MPO-DNA complexes). However the LDG’s NETosis capacity and relation to hypercoagulability was not directly assessed here (165).

Taken together, aberrant NET formation in severe COVID-19 cases may thus be involved in the observed increased incidence of immunothrombosis. Many of the reports about the first COVID-19 patients’ symptomes describe changes in laboratory coagulation parameters, as well as the occurrence of thrombi, and this is associated with a poor prognosis (166, 167). Skendros and colleagues demonstrated TF-bearing NETs formed by blood neutrophils from patients with severe COVID-19 (168). TF expression in the NETs was mediated by the complement factor C5a (168). Furthermore, increased levels of bioactive TF woven into the NETs of neutrophils during sepsis increase thrombin generation and activate (PAR-1 signaling in) platelets (53), so it is very likely that these TF-bearing NETs formed in COVID-19 patients lead to an increased risk of coagulopathy and thrombus formation. In line, COVID-19 plasma was able to induce the production of bioactive TF in cultured lung fibroblasts and this was severely diminished after treatment with DNase I, pointing towards an involvement of circulating NETs in COVID-19 plasma in sustaining the coagulation cascade (169). In order to prove the beneficial effect of NETs dissolution, the authors of this manuscript also conducted a clinical trial with COVID-19 patients with severe respiratory failure. These patients were treated with a combined therapy consisting of inhaled dornase alfa (DNAse), the IL-6 antagonist tocilizumab and the Janus kinase (JAK) 1/2 inhibitor baricitinib on top of standard treatment (dexamethasone and heparin). This therapy proved to significantly reduce in-hospital mortality, intubation rate, as well as overall duration of hospitalization and survival in comparison to standard treatment alone (169). Additionally, C5a may induce NET formation after priming of neutrophils with other cytokines (170), and interestingly it is also raised in plasma levels of COVID-19 patients (171).

In a study including 36 COVID-19 patients, roughly 22% of the individuals developed thrombotic events, but curiously platelet activation biomarkers (P-selectin, soluble P-selectin, platelet-derived microparticles), though elevated, did not correlate with disease severity and thrombosis (151). Instead, NET markers, such as MPO-DNA complexes and H3cit, are significantly higher in patients with thrombotic events and are also associated with disease severity and with the development of ARDS (151, 162). Nonetheless, platelets are undoubtedly active upon SARS-CoV-2 infection. COVID-19 patients have increased plasma levels of CXCL4 and CCL5 (162), two platelet chemokines known to promote NET formation (17, 35). Indeed, platelets of COVID-19 patients seem capable of interacting with SARS-CoV-2 *via* ACE-2-independent mechanisms and upon contact with the virus, increase the secretion of GPVI, CXCL4 and CCL5 *in vitro*, as well as surface expression of P-selectin (172). Thus, platelets are highly activated after contact with the virus and may release pro-NETotic chemokines. Conformingly, subclusters of platelets with a higher reactivity were found, probably contributing to the immunothrombotic events (163), and another group that examined the transcriptional profile of platelets in COVID-19 cases with pneumonia hypothesized based on their data, that infection with SARS-CoV-2 activates circulating platelets in a VWF/GPIb-IX-V dependent manner to propagate immunothrombosis (173). Moreover, platelet-derived extracellular vesicle counts are raised in COVID-19 patients (174), and platelet-derived, as well as endothelial cell-derived microparticles seem to be the cause of endothelial cell death and NETosis here (175). This may have been mediated *via* PtdSer exposure on microparticles, which were demonstrated to induce coagulation (63). Here, it was mandatory for endothelial cell death, neutrophil adhesion and NETosis, since annexin V capping of PtdSer abolished all of these effects (175).

NETs can induce thrombin activation *via* the intrinsic pathway of coagulation by acting on FXII (57), and this might also apply to COVID-19. A reduced DNase activity in the plasma of COVID-19 patients leads to defective NET clearance, and this in turn may lead to prolonged FXII activation in the lungs of COVID-19 patients (176). Besides, Zuo et al. described the development of aPL antibodies in COVID-19 patients, which are linked to NET formation, elevated platelet counts and a more severe respiratory disease phenotype (177). The same group later identified the presence of NET-stabilizing anti-NET autoantibodies in COVID-19 patients (178). Anti-NET autoantibodies also positively correlated with other markers of disease severity, such as D-dimers, neutrophil and platelet counts, and the authors suggested prolonged autoantibody occurrence as a putative source for long-term COVID-19 consequences in survivors (178). Taken together, a reduced DNase activity, FXII-mediated thrombin generation, as well as the occurrence of prothrombotic and NET-stabilizing autoantibodies, seems especially inconvenient in the background of COVID-19 and may substantially increase the risk to develop immunothrombotic complications. CD39, also known as ectonucleoside triphosphate diphosphohydrolase 1 (NTPDase-1), is expressed on platelets and leukocytes and an important regulator of purinergic signaling *via* hydrolysis of proinflammatory extracellular ATP (eATP) and ADP (eADP) to adenosine (179). Purinergic signaling molecules, such as eATP and eADP bind to P2Y_12_ on platelets and induce platelet aggregation and coagulation. Hypoxia- and viral-induced increased CD39 expression on T cells and NK cells in COVID-19 patients with pneumonia was observed by Díaz-García and colleagues (180). Moreover, soluble active CD39 (sCD39) levels in plasma of COVID-19 patients was associated with longer hospitalisation times, necessity of mechanical ventilation, ICU admission, and death, suggesting sCD39 as a marker for disease severity in COVID-19 patients. Elevated eATP and eADP, as well as decreased adenosine plasma levels may be responsible for increased sCD39, with eATP activating the NLRP-3 inflammasome and eADP activating the procoagulant P2Y_12_ receptor on platelets (180).

**Figure 4:**
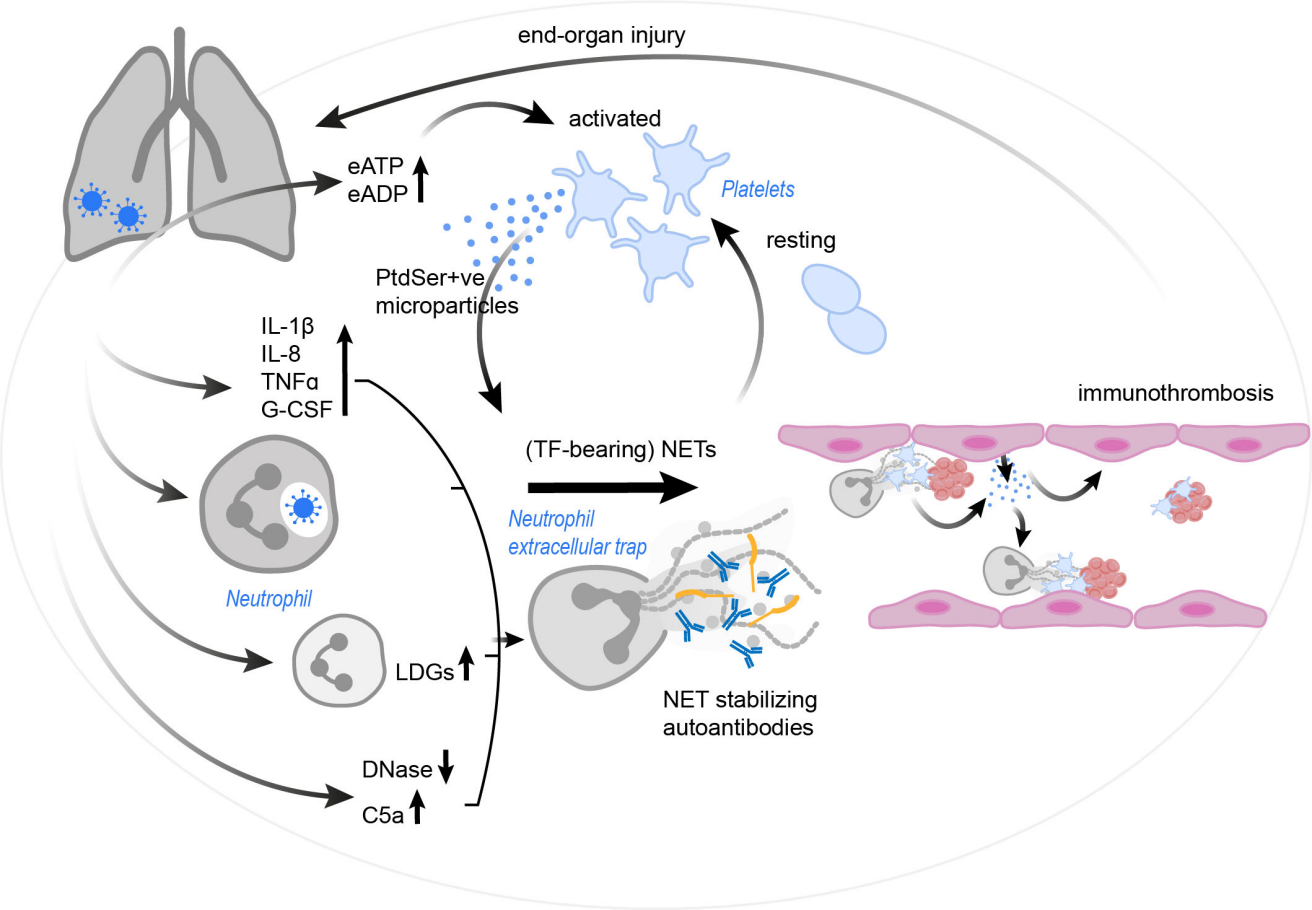
The vicious cycle of platelet-neutrophil interplay contributing to immunothrombosis in COVID-19. SARS-CoV-2 infection results in a drastic increase in pro-inflammatory cytokines and complement C5a, the occurrence of LDGs and a reduction in endogenous DNases. These effects, as well as the virus itself (via currently unknown mechanisms) may trigger and/ or enhance NET formation. Autoantibodies may further stabilize the formed NETs and protect them against removal. NETs in COVID-19 patients were found to have incorporated procoagulant tissue factor. Platelets are activated by the NETs, but also by an increase in eATP and eADP. Platelets might then react with the production of PtdSer+ve microparticles, which in turn may trigger NETosis and drive coagulation. Endothelial cells also release microparticles, stimulating neutrophils for NET formation. The resulting immunothrombosis very likely contributes to significant end-organ damage. C5a, complement C5a; COVID-19, corona virus disease 2019; DNase, deoxyribonuclease; eADP, extracellular adenosine diphosphate; eATP, extracellular adenosine triphosphate; G-CSF, granulocyte-colony stimulating factor; IL, interleukin; LDG, low density granulocyte; NET, neutrophil extracellular trap; PtdSer+ve, phosphatidylserine positive; TF, tissue factor; TNFα, tumor necrosis factor α; SARS-CoV-2, severe acute respiratory syndrome coronavirus-2.

To date it remains unclear, if the NET formation in COVID-19 precedes the activation of platelets or if platelets lead the race towards immunothrombosis and subsequent complications. It might be, that the neutrophils act first by formation of NETs and initiate the subsequent cascade. The observed neutrophilia, as well as the correlation between NET markers and coagulopathy support this framework. Yet, platelets can also be activated by the virus and other components of the immune system. In the end, it is assumable, that the two cascades at least may support each other, since both neutrophils and platelets act as sentinel cells in the lung tissue and thus may get into contact with viral particles, danger-associated molecular patterns (DAMPs) and a cytokine storm, which, following the definition suggested by Fajgenbaum et al., is manifested in COVID-19 patients by elevated circulating cytokine levels and acute systemic inflammatory symptoms with secondary organ dysfunction due to inflammation beyond what would be considered a normal reaction to the virus (181). Given the fact that NETs can cause significant damage in bystander tissue and cells, including the prothrombotic potential, dysregulated NET formation very likely adds to the development of ARDS, lung injury and coagulopathy. Lastly, it also remains elusive what exactly first causes the increase in NET formation, but it is safe to assume, that a combination of the virus infiltration itself, massive cytokine storm, release of EVs with PtdSer exposure, autoantibodies, and activated platelets contribute to this. Another loop might form through the activation of platelets by NET components, such as histones and cathepsin G, which further propagate coagulation and immunothrombosis (**Figure 4**). In a nutshell, once out of control, this path of destruction may develop into a true vicious cycle of destruction, driving the COVID-19 pathology.

Of note, aberrant coagulation appears to play a role not only in acute COVID but also in the high number of patients suffering vascular events in the first year after COVID (182) and at least part of those suffering from “Long-COVID”, which by definition occurs or persists 12 weeks after infection with Sars-CoV-2. Long-COVID patients present with a plethora of symptoms, including often intense fatigue, post-exertional malaise (PEM), dyspnoe, brain fog, and different vegetative symptoms. The formation of fibrin-rich microclots appears to be a potential hallmark of this disease and microclot formation in the lungs that limit oxygen exchange or in the central nervous system could indeed explain many of the above-mentioned symptoms (183, 184). However, Long-COVID research is still in its infancy and the importance of neutrophils and NETs in this context, although very likely high, remains to be elucidated., (**4**)

## 5 Targeting platelet-NET interplay in immunothrombosis

Contemplating NET formation from an evolutional perspective, the dissolution of NETs *in vivo* is executed by endogenous DNases, protecting the host from prolonged damage (185, 186). Hence, in theory, the logical consequence for NET-related pathological mechanisms ought to be the administration of recombinant DNases, and this has been done in many disease models. Likewise, targeting NETs in immunothrombosis must therefore disrupt the network between inflammation and thrombosis. In reality, this rather straightforward solution may not be as simple as anticipated, since neglecting complicated underlying pathophysiological pathways in complex biological systems might be dangerous and offer more problems than protection for the host. As a consequence, localizing the bridges spanning inflammation and thrombosis and identifying the key player is deemed a prerequisite for targeted therapies in platelet-NET interplay-mediated disease onset and progression. To this end, animal models proved to be a helpful tool to gain insight into disease-dependent platelet-NET interplay.

Targeting NETs and/or NET-bound proteins had overall beneficial effects in animal models of venous and arterial thrombosis. DNase infusion protects mice from thrombosis after IVC stenosis (84). Correspondingly, mice from an experimental model of cerebral artery occlusion reacted positive to targeting NETs with recombinant human DNase I to reduce disease burden (80). While DNase in humans may be an intriguing target for acute events, it is currently not an option for long-term prophylactic treatments (for example to prevent cardiovascular events in hypercoagulable patients) as antibody-production against systemic DNase limits its use.

Further successful attempts have been made in targeting PAD4 to inhibit NETosis through abrogation of histone citrullination. PAD4 in mouse models of venous thrombosis may not only present an interesting target because of NETosis prevention, but also possibly because of the putative effects regarding ADAMTS13 inhibition (69). Moreover, PAD4 deficiency in mice did affect NET formation, but not endothelial cell or platelet function, making it all the more attractive regarding unwanted off-target effects (68). In a similar approach, antibodies against citrullinated histones (tACPA) can be successfully applied in different animal models to limit systemic inflammation, for example in a model of rheumatoid arthritis, inflammatory bowel disease or sepsis (187).

A new promising treatment option against NETs is the neonatal NET-inhibitory factor (nNIF) (162). NNIF and nNIF-related peptides are usually found in human umbilical cord blood plasma and efficiently prevent NET formation in neonates blood (188). Mice treated with nNIF upon initiation of brain stroke showed smaller infarct areas and improved outcome, and nNIF generally blocked NET formation and even protected neurons from apoptosis (101). The question is, if nNIF will still be useful, if applied after the insult, or only as a preventive measure. Given the fact that NETs alter the stability of a thrombus, putatively frustrating the success of fibrinolytical therapies, dismantling NETs or inhibiting NET formation simultaneously to thrombolysis may improve the outcome of patients with venous and arterial thrombosis. In a mouse model of photothrombotic stroke, producing a platelet-rich fibrin-free clot, administration of DNase I or Cl-amidine (targeting PAD4), as well as TLR4-deficiency in platelets completely reversed and inhibited thrombus formation, respectively (104). The same group recently showed that administration of DNase I in another murine stroke model, that produces fibrin-rich clots, reduced the infarct size and improved the outcome despite not being able to recanalize the occluded vessel (105). Thus, we suggest that a combined therapy including NET dissolution and fibrinolysis may be a promising approach for future investigations regarding ischemic stroke. Targeting NETs also proved useful in aPL syndrome, where application of DNase reversed NET-mediated thrombin generation *in vitro*, but also application of warfarin (directed against coagulation factors) had protective effects (79). Treatment of mice in an animal model of small intestinal tumor with LMWH, but not the anticoagulant warfarin, resulted in a reduction of hypercoagulation, as well as tumor development and concomitant with this, humans with small intestinal cancer suffering from hypercoagulability did not react to warfarin treatment (143). Since heparin, an anticoagulant, was shown to effectively dismantle NETs *in vitro* (10) and the anticoagulant warfarin did not show any positive effects in the study, it may be more promising to target NET formation as the underlying issue here.

Beyond NETs as a treatment target, platelets may promote NET formation, and platelet-derived HMGB1 was repeatedly identified as key molecule in venous (77, 78), as well as arterial thrombosis (101). Thus, targeting platelet effector mechanisms presents another option in disrupting inflammation/NETosis-induced thrombosis. Inhibiting HMGB1 with BoxA reduced thrombus burden in DVT and improved ischemic stroke outcome (77, 101). However, inhibiting HMGB1 will probably have off-target consequences, as stated by Dyer and colleagues, since it is expressed in various cell types including platelets and neutrophils (78). In addition, the injection of thrombomodulin, a transmembrane glycoprotein with anti-coagulant properties, was recently shown to degrade HMGB1, which in turn inhibited NET formation and the metastasis of pancreatic cancer in mice (189). Thus, reverting the effects of HMGB1 in an inflammatory background may accomplish beneficial results and proved successful in a variety of murine inflammatory disease models, and currently drug options targeting HMGB1 are in discussion (190). In an animal model of influenza infection, where dissolution of NETs failed to improve the outcome of sick animals, targeting thrombin generation or platelet PAR-4 instead reduced NET formation and improved the outcome (44), demonstrating that in the case of platelet-mediated NET formation, these mechanisms may represent better treatment targets. Yet, these findings have to be treated with carefulness, as playing with NETosis in the background of infectious diseases was not always a good option for the host, as it may promote pathogen spreading in some cases (27).

The next step after successful identification of treatment targets and pharmaceuticals would be to translate animal models to humans and design clinical studies. However, up to this day almost no translational progress has been made in any of the platelet-NET driven pathological mechanisms. And even though NETs alone have been identified as putative treatment targets in different inflammatory diseases, there seems to be an astonishing minority of studies targeting NETs and mechanisms of NET formation in humans. Currently, one successfully completed study inhibiting NET formation is listed on clinicaltrials.gov (out of a total of 28 listed studies using the search term “neutrophil extracellular traps”). Here, COPD patients were treated with danirixin, a CXCR2 antagonist preventing neutrophil activation, but there was no reduced NETosis in the sputum observed after danirixin treatment (191). One ongoing study now aims to address the benefit of NETs removal from the plasma of sepsis/sepsis-related acute kidney injury patients through plasmapheresis (NCT04749238). The results of this study could be of special interest for future treatment of sepsis patients. In 2018, an open-label study combining rituximab and belimumab (targeting B-cells) conducted with 16 SLE patients published overall beneficial results regarding NET formation, probably *via* inhibition of immune complex formation initially triggering NETosis (192). The outbreak of COVID-19 has sparked the interest in the topic of NETs as targets, and currently, a plethora of different pharmaceuticals targeting the activation of neutrophils (and thus putatively also NET formation) are found in different stages of clinical trials, and this is reviewed elsewhere (193). Kaiser et al. summarized in a recent review a few studies targeting platelet-neutrophil interactions with antibodies against P-selectin or PSGL-1 (194). Preventing the formation of platelet-neutrophil bonds may be an interesting approach regarding platelet-mediated NET formation, also in the background of other diseases. In summary, the reasons for this overall lack of translational progress may be down to the complicated and not yet fully decoded networks in different diseases and therefore unforeseeable off-target/side-effects, which may improve in the future with more research work done in this field.

## 6 Outlook

After nearly two decades of NETosis in science, a prominent observation when browsing the literature is the inhomogeneity in the methods of NET detection, thus the scientific community could benefit from a standardized approach, as already noticed by plenty of reviewers writing in this research field. Though efforts and advances have been made to understand platelet-driven NET formation, as well as NET-mediated platelet activation and coagulation, the intricate networks interweaving inflammation and thrombosis are still not fully understood. Future work will provide more insight, and also with increasing evidence, translational approaches from animal models to humans may be expedited.

## Author contributions

A-KW reviewed the literature, wrote the manuscript and designed the figures. LE contributed to writing the manuscript. JR conceived and extensively reviewed the manuscript. All authors read and approved the final version of the manuscript. All authors contributed to the article and approved the submitted version.

## Funding

This work was supported by the Deutsche Forschungsgemeinschaft (DFG Grant No. RO4537/4-1, RO4537/5-1 and SFB1450C07 to JR).

## Acknowledgments

We especially thank Nina Knubel for helping with the preparation and creation of the figures. Ann-Katrin Wienkamp is a member of the CiM-IMPRS graduate program.

## Conflict of interest

The authors declare that the research was conducted in the absence of any commercial or financial relationships that could be construed as a potential conflict of interest.

## Publisher’s note

All claims expressed in this article are solely those of the authors and do not necessarily represent those of their affiliated organizations, or those of the publisher, the editors and the reviewers. Any product that may be evaluated in this article, or claim that may be made by its manufacturer, is not guaranteed or endorsed by the publisher.
